# Promoter Hypermethylation of Tumor-Suppressor Genes *p16^INK4a^, RASSF1A, TIMP3*, and *PCQAP/MED15* in Salivary DNA as a Quadruple Biomarker Panel for Early Detection of Oral and Oropharyngeal Cancers

**DOI:** 10.3390/biom9040148

**Published:** 2019-04-12

**Authors:** Chamikara Liyanage, Asanga Wathupola, Sanjayan Muraleetharan, Kanthi Perera, Chamindie Punyadeera, Preethi Udagama

**Affiliations:** 1 Department of Zoology and Environment Sciences, University of Colombo, Colombo 03 00300, Sri Lanka; chamikara1@live.com (C.L.); awathupola@zoology.cmb.ac.lk (A.W.); muraly_3@yahoo.com (S.M.); 2 National Cancer Institute of Sri Lanka, Maharagama 10280, Sri Lanka; kanthiperera3@gmail.com; 3 The School of Biomedical Sciences, Institute of Health and Biomedical Innovation, Queensland University of Technology, Kelvin Grove, QLD 4059, Australia; 4 Translational Research Institute, 37 Kent Street, Woolloongabba, Brisbane, QLD 4102, Australia; chamindie.punyadeera@qut.edu.au

**Keywords:** oral cancer, oropharyngeal cancer, tumor-suppressor genes, promoter hypermethylation

## Abstract

Silencing of tumor-suppressor genes (TSGs) by DNA promoter hypermethylation is an early event in carcinogenesis; hence, TSGs may serve as early tumor biomarkers. We determined the promoter methylation levels of *p16^INK4a^*, *RASSF1A*, *TIMP3*, and *PCQAP/MED15* TSGs in salivary DNA from oral cancer (OC) and oropharyngeal cancer (OPC) patients, using methylation-specific PCR coupled with densitometry analysis. We assessed the association between DNA methylation of individual TSGs with OC and OPC risk factors. The performance and the clinical validity of this quadruple-methylation marker panel were evaluated in discriminating OC and OPC patients from healthy controls using the CombiROC web tool. Our study reports that *RASSF1A*, *TIMP3*, and *PCQAP/MED15* TSGs were significantly hypermethylated in OC and OPC cases compared to healthy controls. DNA methylation levels of TSGs were significantly augmented by smoking, alcohol use, and betel quid chewing, indicating the fact that frequent exposure to risk factors may drive oral and oropharyngeal carcinogenesis through TSG promoter hypermethylation. Also, this quadruple-methylation marker panel of *p16^INK4a^*, *RASSF1A*, *TIMP3*, and *PCQAP/MED15* TSGs demonstrated excellent diagnostic accuracy in the early detection of OC at 91.7% sensitivity and 92.3% specificity and of OPC at 99.8% sensitivity and 92.1% specificity from healthy controls.

## 1. Introduction

Oral and oropharyngeal squamous cell carcinomas (OSCC and OPSCC) are the most common types of head and neck squamous cell carcinoma (HNSCC) ranked as the sixth most common cancer type and the eighth most common cause of cancer death worldwide [1,2]. Annually, cancers in the lip and oral cavity account for more than 130,900 new cases and 74,500 deaths for males in developing countries [2]. Oral cancer (OC) and oropharyngeal cancer (OPC) record a high age-standardized incidence rate (ASIR) in Sri Lanka [3]. Despite the advances in treatment, no significant improvement was witnessed in the five-year survival rate of OC patients in Sri Lanka over the past several decades, where survival remains at 50–55% [4,5]. Tobacco smoking, alcohol consumption, and betel quid chewing are considered as established risk factors for OC and OPC, while there is a dramatic increase in the incidence of OPC attributable to human papillomavirus (HPV) infections [6,7].

Cancer is a disease which evolves from the successive accumulation of genetic and epigenetic alterations [8]. Hypermethylation of cytosine/guanine (CpG) islands in the promoter regions of tumor-suppressor genes (TSGs) cause loss of expression leading to cancer initiation [9]. Research reported that many TSGs are epigenetically silenced by promoter hypermethylation in the OC cell genome [10,11,12,13]. Increasing evidence is mounting that age, race, and tobacco, alcohol, and betel quid carcinogens are capable of inducing promoter hypermethylation in TSGs, which may ultimately induce oral malignancies [14,15,16,17].

Successful screening and surveillance approaches consider the collection of genomic material using minimally invasive approaches. Aberrant DNA methylation can be detected in DNA from serum, sputum, bronchial lavage fluid, urine, and ductal fluids from patients with many different types of cancers [10,18,19,20,21]. Saliva is a complex and important body fluid which is already used for screening cancers in the upper aero-digestive tract, as well as OC and OPC [10,22,23,24,25,26]. Early screening of oral lesions using salivary biomarkers is very promising because saliva is in direct contact with the oral mucosa and cancerous lesions with a low background of inhibitory substances [27]. Methylation array analysis of DNA allowed interrogating cancer-related genes that are specific and sensitive for the early detection of cancer [28]. The methylation-specific polymerase chain reaction (MS-PCR) method provides a highly sensitive, economical, and time-efficient method to detect relatively low concentrations of methylated sequences in salivary rinses [29,30,31,32].

Early detection efforts using molecular markers have the potential to decrease the disease burden and play a significant role in the successful clinical treatment of any cancer [27]. In the current study, we aimed to determine whether promoter methylation of *p16^INK4a^*, *RASSF1A*, *TIMP3*, and *PCQAP/MED15* TSGs in DNA derived from saliva can serve as a diagnostic marker panel in the early detection of OC and OPC. This study focused on two objectives: (1) to determine the promoter methylation levels of TSGs using the MS-PCR method combined with densitometry analysis to assess their association with established and emerging risk factors of OC and OPC, and (2) to evaluate the clinical performance of this methylation marker panel in discriminating OC and OPC subjects from a healthy control cohort using CombiROC analysis.

## 2. Materials and Methods

### 2.1. Ethical Approval

This study received the consent of the Ethics Review Committee (ERC) of the Faculty of Medicine, University of Colombo, Sri Lanka (EC-16-125) for the collection of biological samples and socio-economic and demographic details of OC and OPC patients, and of healthy controls.

### 2.2. Study Subjects and Data Collection

Newly enrolled, treatment-naive cancer patients with primary cancer in the oral cavity (*N* = 54) and oropharynx (*N* = 34) or patients with loco-regional metastasis with oral/oropharyngeal origin who gave voluntary consent were enrolled in this study as case/test subjects, from the National Cancer Institute, Sri Lanka. An interviewer-administered questionnaire was used to collect demographic data and risk factors associated with OC and OPC. Medical information of patients was retrieved from their pathology report with the pathological staging of the tumor and histopathological classification of the tumor grade. Age- and gender-matched healthy individuals (*N* = 60) with no personal history of cancer were recruited as normal healthy control subjects.

### 2.3. Sample Collection

Saliva samples from case and control subjects were collected at baseline. Patients who volunteered to take part in the study were requested to sit in a comfortable upright position. After rinsing the mouth with saline water to remove food debris, subjects were asked to tilt their head down for 5 min to pool saliva in the mouth. A volume of 2 mL of saliva was collected into sterile containers. Dry ice was used to transport sample containers. Samples were centrifuged at 1200× *g* for 10 min at 4 °C to isolate cellular pellet collection [11,33,34]. Sample collection was carried out for 12 months. DNA extraction was performed within seven days of the date of sample collection.

### 2.4. DNA Extraction and Human DNA Confirmation

The DNeasy Blood and Tissue kit (QIAGEN, Hilden, Germany; Cat no: 69504) was used according to the manufacturer’s instructions to extract DNA from cell pellets of saliva samples. PC03 and KM38 primers were used on extracted DNA samples to amplify a 167-bp region of the human β-globin housekeeping gene to confirm the presence of human DNA [35]. PCR-positive samples were used for further analyses.

### 2.5. *HPV-L1* DNA Detection

GP5+/GP6+ primer sequences were used to amplify a 150-bp region of the *HPV-L1* gene [36] (Table 1). The reaction mixture was prepared with the Promega GoTaq Flexi DNA Polymerase PCR kit. Each PCR reaction contained 2.5 mM MgCl_2_, 0.2 mM deoxyribonucleotide triphosphate (dNTP), and 0.5 μM primers. PCR conditions were set up as follows: initial denaturation at 94 °C for 3 min, 40 cycles of denaturation at 94 °C for 45 s, primer annealing at 42 °C for 45 s, primer extension at 72 °C for 45 s, and a final extension at 72 °C for 5 min. Each PCR reaction panel included an HPV-positive control and a negative PCR control. PCR products were visualized on 2% agarose gels with ethidium bromide (EtBr) staining [37].

### 2.6. DNA Bisulfite Conversion

EpiTect Plus DNA Bisulfite Kit (QIAGEN, Hilden, Germany; cat No.: 59124) was used for DNA bisulfite conversion according to the manufacturer’s instructions. The bisulfite reaction mix was prepared at room temperature using 20 μL (1 µg/µL) of thawed DNA. Bisulfite-converted DNA (50 ng/µL) in elution buffer was immediately used for the MS-PCR or stored at −20 °C. DNA purity and the concentrations were assessed using a NanoDrop™-1000 Spectrophotometer (Thermo Scientific, Wilmington, DE, USA).

### 2.7. Target Gene Selection

A panel of four genes with tumor-suppressor activities was selected to examine methylation abnormalities in promoter regions. These TSGs were described as targets for epigenetic silencing in diverse human cancers. Among these, *p16^INK4a^* is involved in cell-cycle control, *RASSF1A* in apoptosis, *TIMP3* in cell invasion, and *PCQAP/MED15* in transcription regulation [10,12,13,26]. These TSGs were already evaluated in other studies, and our plan was to validate them in a different geographical population [22,26,38]. Initially, *p16* promoter hypermethylation was discovered to be involved in the pathogenesis of oral pre-cancerous lesions associated with betel quid chewing in Sri Lanka [15]. Two previous studies reported that *RASSF1A*, *p16*, and *PCQAP/MED15* TSG salivary methylation markers are useful in detecting hypermethylation events in HNSCC patients [11,26]. Similarly, Lim et al. in 2016 showed that a *p16^INK4a^, RASSF1A, TIMP3*, and *PCQAP/MED15* salivary methylation marker panel offers the potential in detecting early-stage HPV-negative HNSCC tumors [39]. Thus, we presumed that the evaluation of promoter hypermethylation of these four selected TSGs would be collectively useful as a diagnostic tool for the early detection of OC/OPC.

### 2.8. Methylation Analysis of TSGs Using MS-PCR Assay

The methylation status of the selected TSGs was detected using the MS-PCR assay for bisulfite-converted DNA [29]. Specificity of each MS-PCR primer pair used in the current study (Table 1) was validated by previous studies [10,11,26,31,39]. Both methylation and unmethylation primer pairs were tested using bisulfite-unconverted DNA to determine their specificity and they were found not to amplify. Unmethylation PCR was used as a normalizer for the methylation PCR. Any sample which showed no unmethylation band was either discarded from the analysis or the PCR was repeated. For each set of MS-PCR, a tumor sample with known hypermethylation was used as a positive control, while DNase/RNase-free distilled water was used for the negative PCR control.

We used a previously reported nested MS-PCR method to detect the promoter CpG methylation of *p16^INK4a^* and *RASSF1A*TSGs [26]. Methylation-independent primers of *p16^INK4a^* and *RASSF1A* TSG*s* were used at 0.5 μM in a standard PCR (25 μL of reaction volume) using Go Taq DNA polymerase (Promega, Promega, Madison, WI, USA) with 3 μL (150 ng) of bisulfite-converted DNA template (diluted either three- or five-fold depending on the DNA concentration) and supplied 1× Go Taq Flexi Buffer, 2.5 mM MgCl_2_, and 0.6 mM dNTPs (Promega-USA). The following cycling conditions were applied for the stage-1 PCR: initial denaturation at 94 °C for 5 min, 35 cycles of denaturation at 94 °C for 30 s, primer annealing at 60 °C for 30 s, primer extension at 72 °C for 30 s, and a final extension at 72 °C for 5 min. To detect unmethylated or methylated alleles for *p16^INK4a^* and *RASSF1A* genes, stage-2 unmethylation and methylation touchdown gradient PCR was carried out using 1 μL from the stage-1 product as the DNA template. Both touchdown gradient PCRs involved an initial denaturation at 94 °C for 5 min, 25 cycles of denaturation at 94 °C for 30 s, primer annealing with temperature decreasing from 64 °C to 56 °C in 2 °C/5-cycle steps, primer extension at 72 °C for 30 s, and a final extension at 72 °C for 5 min.

For both *TIMP3* and *PCQAP/MED15* TSGs, specific methylation and unmethylation primer sets (1 μM) were used in two separate PCR reactions in a standard PCR. For both methylation and unmethylation reactions, a ratio of 25:1 of the total bisulfite-converted DNA template was taken. Methylation and unmethylation PCRs of the *TIMP3* gene consisted of an initial denaturing stage at 95 °C for 5 min, followed by 40 cycles of 30 s at 94 °C, 30 s at 54 °C, and 30 s at 72 °C, and a final extension at 72 °C for 5 min. We used methylation and unmethylation primer sets specific for the CpG islands in the main promoter region of *PCQAP/MED15*gene (*PCQAP5*′). For *PCQAP5*′, both methylation and unmethylation PCRs involved an initial denaturing stage at 95 °C for 5 min, followed by 35 cycles of 30 s at 94 °C, 30 s at 62.5 °C, and 1 min at 72 °C, and a final extension at 72 °C for 5 min.

### 2.9. Agarose Gel Electrophoresis and Determination of Gene Methylation Levels

A volume of 5 μL of each PCR amplicon was visualized on 4% agarose gels. Samples that gave strong consistent bands were exclusively used for the promoter methylation analysis. PCR Fusion SL gel documentation system (VilberLourmat, Marne la Vallee, France) was used to scan gels, and band intensities of each sample were determined using ImageJ software (National Institutes of Health, Bethesda, MD, USA). Finally, the ratio between methylated and unmethylated band intensities was calculated using Microsoft Excel (Microsoft Corporation, Redmond, Washington, DC, USA). Quantification of band intensities was carried out by two independent researchers to minimize observational errors.

### 2.10. Statistical Analysis

Statistical analysis was performed using SPSS version 20 for Windows (IBM-SPSSStatistics, IBM Corporation, Armonk, NY, USA) and Graph-Pad Prism (GraphPad Software, Inc., San Diego, CA, USA) software packages. Comparisons of normally distributed variables of independent samples were performed using the *t*-test. Since methylation levels were not normally distributed, non-parametric Mann–Whitney U test was used to compare methylation levels of normal healthy controls with those of OC and OPC patients. Chi-square test and binary logistic regression assessed the relationship (combined effect and the independent effect, respectively) between the presence of OC/OPC and predictor variables/risk factors. Calculated odds ratios (OR) measured the risk of developing OC and OPC. Wald statistic was used to determine parameter significance (*p*-value) in logistic regression models. All statistical tests were two-tailed, and significant differences between two categorical variables were marked with * *p* < 0.05, ** *p* < 0.001, and *** *p* < 0.0001.

CombiROC web tool (http://CombiROC.eu) was used to determine the clinical performance of these four methylation markers in discriminating OC and OPC subjects from healthy controls [40]. Optimal marker combinations and their clinical performance were determined through the combinatorial analysis of the receiver operating characteristic (ROC) curves provided by the CombiROC tool. The 10-fold cross-validation (CV) step was used to attain a reliable estimation of the clinical performance of the best marker combination [40]. This step is crucial as it could avoid the risk of over-fitting and show how well the panel translates into clinical diagnosis. Analyzing the statistical significance of the area under the curve (AUC) value is imperative, as the CV procedure could generate over-optimistic results. Therefore, permutation tests were performed to assess the statistical significance of AUC values generated by each ROC curve analysis.

## 3. Results

### 3.1. Population Characteristics of the Study Cohorts

Both patient cohorts and controls were found comparable in age and gender. Mean age of OC and OPC subjects and of normal healthy subjects was 62 ± 12.5, 62 ± 10.1, and 60 ± 7.1 years, respectively. A male preponderance was evident in both OC (90.7%) and OPC (94.1%) cohorts (Table 2). A majority of the OC (79.7%) and OPC (91.2%) patients were smokers, where most of them consumed more than five cigarettes daily (Table 2). Higher levels of alcohol consumption (OC = 74.1%; OPC = 91.2%) and betel quid chewing (OC = 77.8%; OPC = 76.4%) were recorded in patient cohorts, where a majority of them were habitual consumers for more than 25 years of their lifespan (Table 2).

Regarding the primary tumor site of cancer patients, most of the OC cases were cancers on the front two-thirds of the tongue (48.1%), while cancers on the back wall of the throat were common in OPC cases (44.1%). In addition, grade 1 (well differentiated) tumors were common among cancer patients (OC = 44.4%; OPC = 50.0%) compared to other tumor grades. Although cancer tumor//node/metastasis (TNM) stage information was unavailable for all patients, the majority of the recruited patients (OC = 42.6%; OPC = 38.2%) were in advanced stages of cancer development (Table 2).

### 3.2. *HPV-L1* Analysis

**Figure 1:**
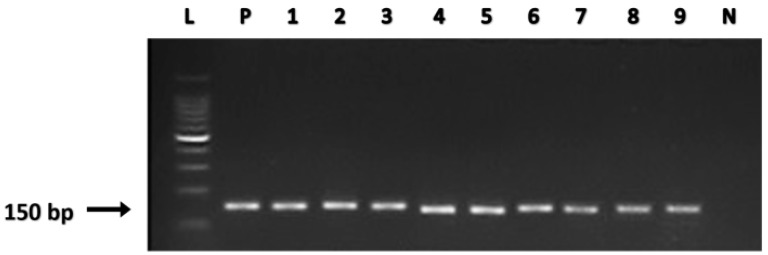
Agarose gel electrophoresis of *HPV-L1* analysis. L: 100-bp DNA ladder with 500-bp marker; P: positive control; N: negative PCR control. Samples 1–5: oral cancer (OC) patient samples; Samples 6–8: oropharyngeal cancer (OPC) patient samples; Sample 9: healthy control sample.

Detecting the presence of the *HPV-L1* in salivary DNA samples provides an accurate output on high-risk HPV strains [36]. In this study, the *HPV-L1* was detected in five OC subjects, three OPC subjects, and a single subject from the normal healthy control cohort (Figure 1).

### 3.3. Combined and Independent Effect Assessment of Etiologic Agents of OC and OPC

Combined and independent effects of the risk factors of OC and OPC are summarized in Table 3. Examination of the effects of established risk factors on OC and OPC revealed that smoking, alcohol use, and betel quid chewing had significant combined effects for both cancer types. Smoking demonstrated the strongest combined effect for OC (OR = 7.3 (95% confidence interval (CI) = 2.8–18.6)), as well as for OPC (OR = 19.2 (95% CI = 4.7–89.8)). In addition, a substantial independent effect was also noted to be driven by smoking for both OC (OR = 7.8 (95% CI = 2.1–28.4)) and OPC (OR = 20.8 (95% CI = 2.4–178.2)). Although the combined effect of HPV infection was not significant for both cancer types, it exerted a substantially high independent effect for OPC (OR = 19.6 (95% CI = 1.0–146.4)) compared to OC (OR = 6.7 (95% CI = 0.6–123.8)) development.

### 3.4. Comparative DNA Methylation Analysis of Individual TSGs

**Figure 2:**
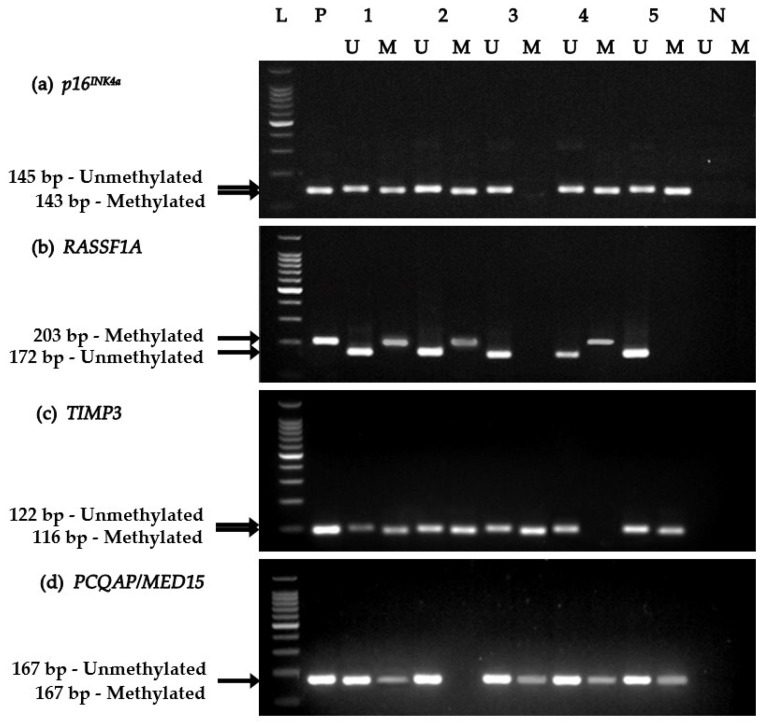
Agarose gel electrophoresis of methylation-specific PCR (MS-PCR) analysis of tumor-suppressor genes (TSGs). L: 100-bp DNA ladder with 500-bp marker; P: positive control; U: unmethylated amplicon; M: methylated amplicon; N: negative PCR controls (for unmethylation and methylation PCR). Samples 1–3: OC patient samples; Samples 4 and 5: OPC patient samples.

**Figure 3:**
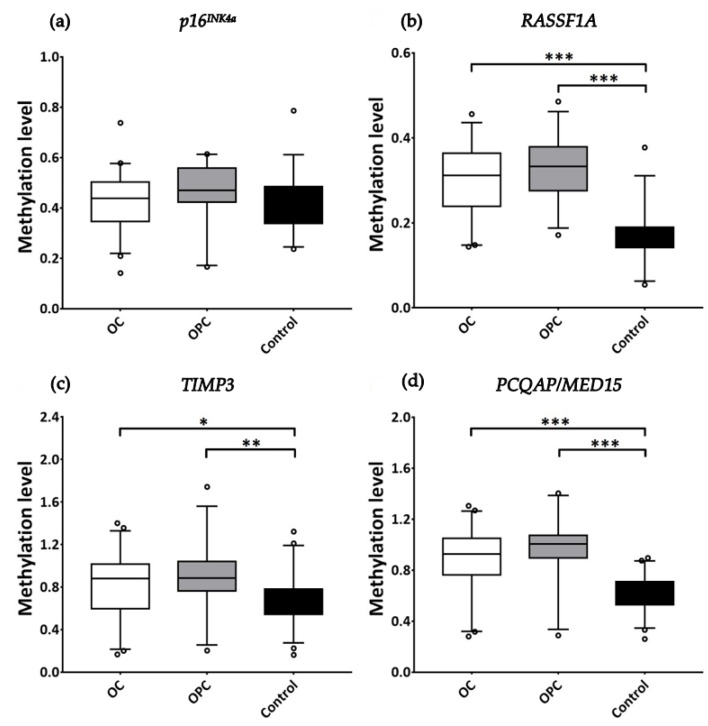
Hypermethylation profiles of individual TSGs among OC, OPC, and normal healthy control cohorts. Whisker-box plots were drawn (on 5–95% percentile) for the methylation signatures of (**a**) *p16^INK4a^*, (**b**) *RASSF1A*, (**c**) *TIMP3*, and (**d**) *PCQAP/MED15* in the saliva collected from OC patients (*N* = 54), OPC patients (*N* = 34), and normal healthy controls (*N* = 60), analyzed using Mann–Whitney U test. Significant difference in the promoter methylation level between two cohorts is marked with * *p* < 0.05, ** *p* < 0.001, or *** *p* < 0.0001.

Agarose gels analyzed for the detection of promoter hypermethylation events for all four TSGs are presented in Figure 2. Salivary DNA promoter methylation levels of TSGs were comparatively analyzed among the three study cohorts (Figure 3). Accordingly, only *RASSF1A*, *TIMP3*, and *PCQAP/MED15* TSGs showed significant promoter methylation (hypermethylation) in saliva collected from OC subjects (*p* < 0.0001, *p* < 0.05, and *p* < 0.0001, respectively) and from OPC subjects (*p* < 0.0001, *p* < 0.001, and *p* < 0.0001, respectively), compared to normal healthy controls. Conversely, there was no significant difference in promoter methylation levels between OC subjects and OPC subjects, for any of the four TSGs examined.

### 3.5. Association between Promoter Methylation of TSGs and Clinicopathological Parameters of OC and OPC

Clinicopathological variables of OC and OPC patients and their exposure to risk factors were compared with the promoter methylation levels of individual TSGs (Table 4). Accordingly, promoter methylation of TSGs indicated no significant association with respect to age and gender. However, a significant promoter hypermethylation was detected in *p16^INK4a^* and *RASSF1A* TSGs (*p16^INK4a^*: *p* < 0.05; *RASSF1A*: *p* < 0.05) in saliva collected from smokers and alcohol consumers, compared to non-consumers (Table 4). Also, betel quid chewers demonstrated significant promoter hypermethylation of all four TSGs (*p16^INK4a^*: *p* < 0.05; *RASSF1A*: *p* < 0.05; *TIMP3*: *p* < 0.001; and *PCQAP/MED15*: *p* < 0.05) compared to non-consumers (Table 4). Further, we found that *p16^INK4a^, TIMP3*, and *PCQAP/MED15* TSGs were significantly hypomethylated (*p* < 0.001, *p* < 0.001, and *p* < 0.001, respectively) in saliva of HPV-positive subjects compared to HPV-negative subjects (Table 4).

A significant *p16^INK4a^* and *RASSF1A* promoter hypermethylation (*p* < 0.001 and *p* < 0.05, respectively) was observed in advanced OC stages, compared with early/less advanced OC stages (Table 4). In addition, substantial promoter hypermethylation was noted between *p16^INK4a^* and *RASSF1A* TSGs (*p* < 0.05 and *p* < 0.05, respectively) in high-grade (grades 3 and 4) OC tumors compared to low-grade (grades 1 and 2) OC tumors. For OPC, *p16^INK4a^*, *RASSF1A*, and *TIMP3* TSGs were significantly hypermethylated (*p* < 0.05, *p* < 0.05, and *p* < 0.05, respectively) in high-grade OPC tumors compared to low-grade OPC tumors (Table 4).

### 3.6. Performance of the Methylation Marker Panel in Discriminating OC and OPC from Healthy Controls

**Figure 4:**
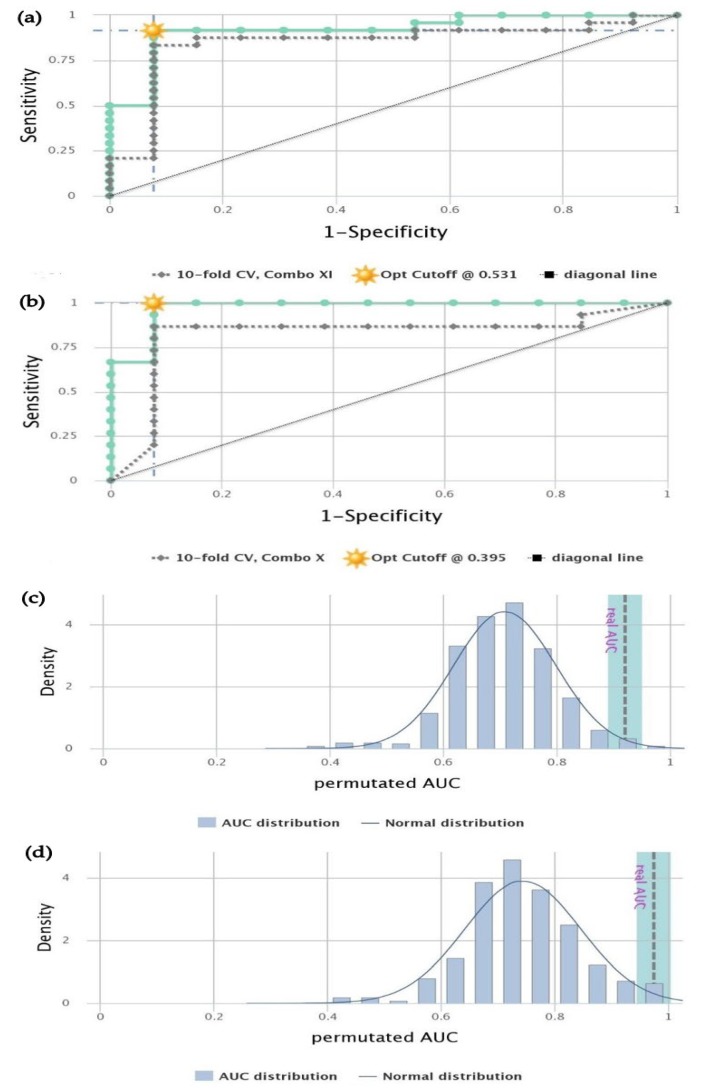
Performance of the quadruple-methylation marker panel. CombiROC curve analysis of the marker panel in discriminating (**a**) OC patients and (**b**) OPC patients from normal healthy controls. Green bar: receiver operating characteristic (ROC) curve of the marker panel for the whole cohort. Gray dotted bar: 10-fold cross-validation (CV) test of the marker panel. Diagonal line: reference line with zero discriminating power (0.5 sensitivity and specificity). Density distribution of permutated area under the curve (AUC) values compared to the normal distribution, illustrating the significance of real AUC value generated for discriminating (**c**) OC subjects and (**d**) OPC subjects from healthy controls.

The CombiROC curve analysis identified the best performing marker combination by evaluating its clinical performance in discriminating OC, OPC, and healthy control cohorts (Table 5). Intriguingly, the marker combination of all four markers (quadruple-methylation marker panel) performed well in discriminating OC patients from healthy controls with an AUC of 0.92 and accuracy (ACC) of 0.92, with a sensitivity of 91.7% and 92.3% specificity (Table 5; Figure 4a). Furthermore, this marker panel performed exceptionally well with an AUC of 0.97 and ACC of 0.96, with a sensitivity of 99.8% and 92.1% specificity when discriminating OPC patients from healthy controls (Table 5; Figure 4b). Next, the performance of the quadruple-methylation marker panel was investigated using a 10-fold CV and permutation test, aiming for the effective clinical validation of the marker panel. Results of the CV procedure suggest that the quadruple-methylation marker panel is a perfect fit for the diagnosis of OC and OPC patients from healthy controls, as the overall accuracy, sensitivity, and specificity were least affected by the imposed likelihood (Table 5; Figure 4a,b). In addition, we obtained the “real AUC value” outside the permutated AUC distribution, which signified the high validity of this marker panel in discriminating OC and OPC subjects from normal healthy controls (Figure 4c,d).

## 4. Discussion

This study evaluated cohorts of OC and OPC patients and matched healthy controls to determine the utility of the MS-PCR approach combined with a densitometry analysis to assess promoter methylation levels of *p16^INK4a^*, *RASSF1A*, *TIMP3*, and *PCQAP/MED15* TSGs in saliva-derived DNA. Conferring to the high diagnostic accuracy detected, the strong potential of this quadruple-methylation marker panel in discriminating OC and OPC patients from healthy controls was clearly evident.

TSGs analyzed in our study were found to be associated with common oncogenic transformation pathways and cellular functions that are frequently dysregulated in many cancers including OC and OPC. The *p16^INK4a^* gene encodes for a cyclin-dependent kinase 4 (CDK 4) inhibitor which regulates the retinoblastoma (Rb) pathway and arrests the cell cycle [41]. Hence, its inactivation may lead to disruption of cell cycle control ultimately inducing tumorigenesis in various cancers including OC [42,43,44,45,46]. However, no significant *p16^INK4a^* promoter methylation was observed in both OC and OPC salivary rinses, although this gene was reported to be highly methylated in previous HNSCC studies [15,26,44,47,48]. A recent study of OSCC patients in a south Indian population reported significant *p16* promoter hypermethylation (38%) [49]. This contradiction of detecting different methylation frequencies can be explained by the differences in sampling methods, the sensitivity of the detection method, location and length of investigated CpG repeats, and cohort composition.

Ras association domain family 1 isoform A (RASSF1A) acts as a downstream negative effector of the Ras protein which induces growth arrest of cells (apoptosis) [50]. Recent evidence suggests that *RASSF1A* expression is dependent on angiogenic signaling events during cancer progression [51]. It is well documented that the *RASSF1A* TSG is frequently inactivated in primary oral tumors by de novo methylation of promoter CpG islands, subsequently triggering OC initiation [26,39,52]. Significant *RASSF1A* promoter hypermethylation detected by our study in both OC and OPC salivary rinses suggests its strong association with OC and OPC risk.

Tissue inhibitor of metalloproteinases (TIMPs) is known as an inhibitor of cellular invasion, metastasis and angiogenesis [53]. Loss of TIMP expression by *TIMP3* TSG promoter hypermethylation is speculated specifically with HNSCC neoplastic evolution [38,54]. Our study reiterates previous reports that evidence the positive correlation between *TIMP3* promoter hypermethylation in oral malignancies [12,38,55,56].

Similarly, we observed a substantial *PCQAP/MED15* hypermethylation in both OC and OPC, which is in line with previous studies on HNSCC [11,39]. Although *PCQAP/MED15* methylation in HNSCC was identified and validated recently, it is frequently implicated in prostate and endometrial cancer etiologies [11,42]. The PC2 glutamine/Q-rich-associated protein (PCQAP) is identified as a transcriptional co-activator mediator, responsible for the transcriptional regulation of ligand-activated proteins which plays a pivotal role in cellular regulation, proliferation, and differentiation [11,57].

Age- and sex-specific differences in promoter hypermethylation of *p16^INK4a^* and *RASSF1A* TSGs were previously reported [58]. Conversely, no direct association was observed between TSG promoter methylation with age and sex in both cancer types, in agreement with a few previous reports [39]. It is well known that *p16^INK4a^* and *RASSF1A* promoter methylation status is strongly correlated with the cancer stage [59,60]. By confirming these findings, our study established a significant *p16^INK4a^* and *RASSF1A* promoter hypermethylation in advanced stage of OC subjects, suggesting that these alterations may occur late in the carcinogenesis of the oral cavity. Also, we found significant promoter hypermethylation in high tumor grades of OC for *p16^INK4a^* and *RASSF1A* and of OPC for *p16^INK4a^*, *RASSF1A*, and *TIMP3*, suggesting the correlation between promoter hypermethylation of tumor-related genes and poor prognosis of OC and OPC [26,61,62].

Our results strengthened the notion that smoking and alcohol consumption are strong predictors of *p16^INK4a^* and *RASSF1A* promoter hypermethylation [63,64,65]. Carcinogens in tobacco smoke could drive genetic and epigenetic mutations in frequently exposed tissues [66]. Tobacco smoking also induces DNA methyltransferase (DNMT) activity, thereby causing de novo methylation on disposed loci on a gene-specific basis [67,68]. Significant promoter hypermethylation observed in OPC subjects compared to OC subjects may be attributed to the higher independent risk of smoking observed in OPC subjects. In addition, it is well established that betel nut, as well as betel quid, with or without tobacco, is carcinogenic to HNSCC [69,70]. Betel quid chewing is critically associated with high-risk pre-cancerous oral lesions [15,71]. Furthermore, recent studies report that silencing of *RASSF1A* and *p16^INK4a^* gene expression by promoter hypermethylation may play a critical role in betel-associated oral carcinogenesis [15,72]. In agreement with prior findings, we describe a strong association between betel quid chewing and promoter hypermethylation of all four TSGs studied.

HPV is recognized as an additional independent risk factor for the development of OSCC, particularly OPSCC, confirmed by our risk analysis [73,74,75]. In addition, we report that *p16^INK4a^, TIMP3* and *PCQAP/MED15* promoter regions were hypomethylated in HPV-positive cases compared to HPV-negative cases. This result seems to be paradoxical since HPV infection appears to have a greater association with promoter hypermethylation of TSGs, due to the over-expression and increased DNMT activities induced by HPV oncoproteins (E7) [76,77,78]. This contradiction may be caused by differences in sampling methods, ethnic origin of the subjects, and by the HPV genotype.

It follows that lifestyle factors contribute to tumorigenesis through revocable epigenetic dysregulations, thus holding great promise in disease prevention and treatment [79]. In addition, methylation profiles of TSGs in combination with clinicopathological characters would be useful in predicting the behavior of OC and OPC. The significance of the association between clinicopathological characteristics and promoter methylation of TSGs evaluated by the current study led us to hypothesize the degree of effect of risk factors that may have affected the performance of the quadruple-methylation marker panel. For instance, the higher AUC and sensitivity in detecting OPC patients compared to OC patients may be attributed to the substantial effect of risk factors on the development of OPC compared to OC.

Early detection of cancer, at a stage where it is localized and treatable, will substantially reduce mortality owing to the disease. For this reason, diagnostic tumor biomarkers are now an imperative field pursued in biomedical engineering [80]. It is now well established that individual biomarkers may not be sufficiently accurate in diagnosing tumors, as heterogeneity of individual tumors is triggered by specific molecular alternations in different tumor-related genes [81]. Therefore, combined biomarker panels are developed to attain significant specificity and sensitivity values for effective translation into diagnostic settings [22,54]. Our results suggest that *p16^INK4a^* methylation alone should not be considered as a tumor marker for OC or OPC, since no significant methylation was found in normal and tumor samples. However, hypermethylation of *p16* was found to be a promising diagnostic and prognostic biomarker for recurrence-free survival of OC and OPC [82,83,84]. Hence, CombiROC analysis allowed us to develop a better performing combination of independent methylation markers, by lowering the risk of missing *p16* marker. We defined the high performance of this quadruple-methylation marker panel which outperformed the previous individual markers and other marker combinations substantiated by previous studies in different ethnic groups [26,39,54,81,85]. We further strengthened our study by analyzing the performance, robustness, and consistency of the marker panel, through cross-validation and permutation tests, as such features of this panel would be decidedly useful as a robust diagnostic and screening tool.

The high performance of this marker panel prompts one to study its prognostic potential which could be useful during disease surveillance following treatment or with high-risk cohorts, such as smokers and alcohol users. Notably, evaluating the accuracy of genetic screening tests for oral HPV infections is a promising area of research. However, a genetic screening method for oral HPV infections is yet to be approved by the Food and Drug Administration (FDA); hence, it is rarely practiced in developing countries. A previous study reported the high accuracy of a methylation marker panel in discriminating HPV-positive HNSCC cases from HPV-negative counterparts [39]. Given the low prevalence of HPV-positive OC/OPC cases in our study, the current marker panel was not assessed for its performance in discriminating HPV-positive cancer cases from their HPV-negative counterparts. The low prevalence of HPV in our analysis (9.1%) is probably explained by the abundance of squamous cell carcinomas of the oral cavity (61.4%) among our samples, in agreement with previous reports [86,87].

Since its introduction, the MS-PCR method progressively gained favor as a preferred tool for detecting the DNA methylation status of CpG islands. Nevertheless, some studies showed that it was prone to overestimate methylation prevalence, leading to a high number of false positives (i.e., low specificity) due to incomplete bisulfite conversion [88,89]. Also, it cannot distinguish the methylation status between normal epithelium and tumor cells. This explains the different methylation levels observed in the current study compared to available literature. A previous study, validating TSG methylation markers for diagnosing OSCC, showed that quantitative methylation-specific PCR analyses (Q-MSP) may be a better choice in improving the level of detection. Alternatively, pyrosequencing technology emerged as a robust and versatile platform in global DNA methylation quantification, which may overcome the limitations of MSP-based methods [90,91]. Several studies evaluated both the accuracy and precision of pyrosequencing for the analysis of DNA methylation in heterogeneous mixtures of tumor DNA samples [92,93].

Our work clearly emphasizes the need for assessing other promising genes in saliva to improve the sensitivity and specificity of this current marker panel. The optimized marker panel will finally be evaluated for performance in prospective trials to depict its clinical efficacy. Validation of this panel is expected to involve screening of high-risk populations followed by clinical assessment using biopsy tests for individuals whose methylation profile expresses the highest risk for OC or OPC. Our findings highlight the potential of this marker panel comprising epigenetically silenced TSGs in saliva, which can be used for the earliest possible identification of OC/OPC incidence, permitting careful monitoring to guide immediate intervention and further evaluation.

## 5. Conclusions

We report that frequent exposure to established risk factors such as smoking, alcohol use, and betel quid chewing may drive oral and oropharyngeal carcinogenesis through promoter hypermethylation of TSGs. Essentially, we established the exceptional diagnostic accuracy of the methylation marker panel consisting of *p16^INK4a^*, *RASSF1A*, *TIMP3*, and *PCQAP/MED15* TSGs in the early diagnosis of OC and OPC.

## Figures and Tables

**Table 1:** Primer sequences used in the study.

Gene	Primer Sense (5′–3′)	Primer Antisense (5′–3′)	PCR Product Size (bp)
*β-globin*	PC03: ACACAACTGTGTTCACTAGC	KM38: TGGTCTCCTTAAACCTGTCTTG	167
*HPV-L1*	GP5+: TTTGTTACTGTGGTAGATACTAC	GP6+: GAAAAATAAACTGTAAATCATATT	150
*p16^INK4a^* (MI)	GAGGAAGAAAGAGGAGGGGTTG	ACAAACCCTCTACCCACCTAAATC	274
*p16^INK4a^* (M)	GAGGGTGGGGCGGATCGC	GACCCCGAACCGCGACCG	143
*p16^INK4a^* (U)	TTATTAGAGGGTGGGGTGGATTGT	CAACCCCAAACCACAACCATAA	145
*RASSF1A* (MI)	GGAGGGAAGGAAGGGTAAGG	CAACTCAATAAACTCAAACTCCC	260
*RASSF1A* (M)	GGGGGTTTTGCGAGAGCGC	CCCGATTAAACCCGTACTTCG	203
*RASSF1A* (U)	GGTTTTGTGAGAGTGTGTTTAG	ACACTAACAAACACAAACCAAAC	172
*TIMP3* (M)	GCGTCGGAGGTTAAGGTTGTT	CTCTCCAAAATTACCGTACGCG	116
*TIMP3* (U)	TGTGTTGGAGGTTAAGGTTGTTTT	ACTCTCCAAAATTACCATACACACC	122
*PCQAP 5*′ (M)	GTTTTGTGATTGAGGYGGCGGC	AAAAATCCCACAATCCAACCC	167
*PCQAP 5*′ (U)	GTTTTGTGATTGAGGYGGTGGT	AAAAATCCCACAATCCAACCC	167

MI: methylation-independent primers; M: methylation primers; U: unmethylation primers.

**Table 2:** Socio-demographic and tumor characteristics of the study cohorts.

	OC (*N* = 54)	OPC (*N* = 34)	Healthy Controls (*N* = 60)
**Demographic characteristics**			
**Mean age**	62	62	60
<50	7 (13.0)	3 (8.8)	1 (1.6)
50–59	11 (20.3)	13 (38.2)	29 (48.3)
>60	36 (66.7)	18 (53.0)	30 (50)
**Gender**			
Male	49 (90.7)	32 (94.1)	55 (91.6)
Female	5 (9.3)	2 (5.9)	5 (8.4)
**Smoking**			
Cigarettes/day smoked			
Non-smokers	11 (20.3)	3 (8.8)	39 (65.0)
1 to 5	14 (26.0)	11 (32.4)	8 (13.3)
>5	29 (53.7)	20 (58.8)	13 (21.6)
**Alcohol use**			
Number of years in use			
Non-drinkers	14 (25.9)	3 (8.8)	31 (51.6)
1–25	14 (25.9)	12 (35.3)	10 (16.6)
>25	26 (48.2)	19 (55.9)	19 (31.6)
**Betel quid chewing**			
Number of years in use			
Non-consumers	12 (22.2)	8 (23.5)	41 (68.3)
1–25	16 (29.6)	9 (26.5)	7 (11.6)
>25	26 (48.2)	17 (50.0)	12 (20)
**HPV infection**			
HPV Positive	5 (9.2)	3 (8.8)	1 (1.6)
HPV Negative	49 (90.8)	31(91.2)	59 (98.4)
**Tumor characteristics**			
**Anatomic site**			
Lips	2 (3.7)		
Tongue (Front 2/3)	26 (48.1)		
Hard palate	1 (1.9)		
Buccal mucosa	21 (38.9)		
Mouth floor	3 (5.5)		
Retromolar	1 (1.9)		
Tongue (Back 1/3)		0 (0)	
Soft palate		7 (20.6)	
Tonsillar pillar		12 (35.3)	
Back wall of the throat		15 (44.1)	
**Tumor grade**			
Well differentiated (1)	24 (44.4)	17 (50.0)	
Moderately differentiated (2)	7 (13.0)	5 (14.8)	
Poorly differentiated (3)	0 (0)	2 (5.9)	
Undifferentiated (4)	19 (35.2)	6 (17.6)	
Unknown	4 (7.4)	4 (11.7)	
**Tumor stage**			
Early stage (I, II)	8 (14.8)	2 (5.9)	
Advanced stage (III, IV)	23 (42.6)	13 (38.2)	
Unknown	23 (42.6)	19 (55.9)	

OC: oral cancer; OPC: oropharyngeal cancer; HPV: human papillomavirus. *N*: Total number of subjects in each cohort.

**Table 3:** Combined and independent effects of established and emerging risk factors of OC and OPC.

Cancer Type	Predictor Variable/Risk Factor	Crude OR (95% CI)	*p*-Value ^a^	Adjusted OR^R^ (95% CI)	*p*-Value ^b^
OC	Smoking	7.3 (2.8–18.6)	<0.0001 ***	7.8 (2.1–28.4)	<0.05 *
	Alcohol use	3.1 (1.2–7.3)	<0.05 *	0.6 (0.1–2.0)	0.385
	Betel quid chewing	7.1 (3.0–19.2)	<0.0001 ***	5.7 (2.2–14.3)	<0.05 *
	HPV infection	6.0 (0.6–140.9)	0.07	6.7 (0.6–123.8)	<0.05 *
OPC	Smoking	19.2 (4.7–89.8)	<0.0001 ***	20.8 (2.4–178.2)	<0.05 *
	Alcohol use	11.0 (2.7–51.0)	<0.0001 ***	0.8 (0.1–6.7)	0.854
	Betel quid chewing	7.0 (2.4–20.7)	<0.0001 ***	3.9 (1.2–12.4)	<0.05 *
	HPV infection	5.7 (0.4–148.9)	0.133	19.6 (1.0–146.4)	<0.05 *

OR: odds ratio; CI: confidence interval. Non-consuming/non-infected group was selected as the reference group for each predictor variable/risk factor. Combined effects of risk factors were assessed using crude OR values derived by chi-square test ^a^, while independent effects were assessed using adjusted OR values derived by binary logistic regression analysis ^b^. Adjusted OR^R^: adjusted for smoking, alcohol use, betel quid chewing, and HPV infection. Significant effect of each predictor variable is marked with * *p* < 0.05 or *** *p* < 0.0001.

**Table 4:** Association of promoter hypermethylation of tumor-suppressor genes (TSGs) with demographic factors, risk factors, and clinicopathological characteristics.

Variable	Category		*N*	*p16^INK4a^*		*RASSF1A*		*TIMP3*		*PCQAP/MED15*	
				Meth	*p*-Value ^b^	Meth	*p*-Value ^b^	Meth	*p*-Value ^b^	Meth	*p*-Value ^b^
Age	≥62 ^a^		28	13	0.455	12	0.244	18	0.414	21	0.550
	<62 ^a^		60	58		54		52		52	
Gender	Male		80	63	0.053	61	0.133	69	0.152	66	0.164
	Female		8	5		4		5		5	
Smoking	Consumers		74	57	<0.05 *	58	<0.05 *	61	0.199	62	0.240
	Non-consumers		14	11		7		13		9	
Alcohol use	Consumers		71	56	<0.05 *	56	<0.05 *	61	0.665	60	0.060
	Non-consumers		17	12		9		13		11	
Betel quid Chewing	Consumers		68	51	<0.05 *	52	<0.05 *	61	<0.001 **	57	<0.05 *
	Non-consumers		20	17		13		13		14	
*HPV-L1*	HPV-positive		8	6	<0.001 **	6	0.061	8	<0.001 **	7	<0.001 **
	HPV-negative		80	62		54		66		64	
Tumor grade	OC	Grade 3, 4	19	14	<0.05 *	13	<0.05 *	15	0.311	16	0.289
		Grade 1, 2	31	25		24		28		27	
	OPC	Grade 3, 4	8	6	<0.05 *	5	<0.05 *	4	<0.05 *	2	0.146
		Grade 1, 2	22	17		17		21		21	
Tumor stage	OC	Stage (III, IV)	23	18	<0.001 **	17	<0.05 *	17	0.233	19	0.712
		Stage (I, II)	8	7		6		7		6	
	OPC	Stage (III, IV)	13	8	0.525	10	0.930	10	0.076	11	0.964
		Stage (I, II)	2	2		2		2		1	

^a^ Mean age; ^b^ Mann–Whitney U test; *N*: total number of patients in each category; Meth: number of patients with methylated gene promoter regions. A significant difference in the promoter methylation level between two categories is indicated as * *p* < 0.05 or ** *p* < 0.001.

**Table 5:** CombiROC curve analyses and validation tests of the quadruple-methylation marker panel.

	AUC	ACC	Error Rate (1 − ACC)	SE%	SP%	PPV%	NPV%
(a) Performance in discriminating OC from healthy controls							
Whole cohort	0.92	0.92	0.08	91.7	92.3	95.7	85.7
10-fold CV	0.85	0.87	0.14	83.3	92.3		
Permutated models	0.71	0.71	0.29	69.7	76.0		
(b) Performance in discriminating OPC from healthy controls							
Whole cohort	0.97	0.96	0.04	99.8	92.1	93.7	98.6
10-fold CV	0.82	0.89	0.11	86.7	92.3		
Permutated models	0.60	0.66	0.33	65.1	68.7		
(c) Performance in discriminating OC from OPC							
Whole cohort	0.65	0.72	0.28	91.7	40.0	61.2	53.3
10-fold CV	0.60	0.56	0.44	33.3	93.3		
Permutated models	0.70	0.70	0.30	68.8	73.9		

Clinical performance of the quadruple-methylation marker panel in discriminating (a) OC from healthy controls, (b) OPC from healthy controls, and (c) OC from OPC was determined with respect to all key parameters. AUC: area under the curve; ACC: accuracy; SE: sensitivity; SP: specificity; PPV: positive predictive value; NPV: negative predictive value.
